# Various Morphologies of Graphitic Carbon Nitride (g-C_3_N_4_) and Their Effect on the Thermomechanical Properties of Thermoset Epoxy Resin Composites

**DOI:** 10.3390/polym16131935

**Published:** 2024-07-06

**Authors:** Dina Al Mais, Samir Mustapha, Yasmine N. Baghdadi, Kamal Bouhadir, Ali R. Tehrani-Bagha

**Affiliations:** 1 B. & W. Bassatne Department of Chemical Engineering and Advanced Energy‚ American University of Beirut, Beirut P.O. Box 110236, Lebanon; dya08@mail.aub.edu; 2 Department of Mechanical Engineering‚ American University of Beirut, Beirut P.O. Box 110236, Lebanon; 3 Department of Chemical Engineering‚ Imperial College London‚ London SW7 2BX‚ UK; y.baghdadi20@imperial.ac.uk; 4 Department of Chemistry‚ American University of Beirut, Beirut P.O. Box 110236, Lebanon; kb05@aub.edu.lb; 5 School of Chemical Engineering‚ Aalto University‚ 02150 Espoo, Finland

**Keywords:** epoxy resin, g-C_3_N_4_, mechanical properties, thermal properties, fracture toughness

## Abstract

This research aims to highlight the importance of diverse forms of graphitic carbon nitride (g-C_3_N_4_) as strengthening elements in epoxy composites. It explores the influence of three different forms of g-C_3_N_4_ and their concentrations on the mechanical properties of the epoxy composites. Various characterization techniques, such as scanning electron microscopy (SEM), dynamic light scattering (DLS), thermogravimetric analysis (TGA), and Fourier-transform infrared spectroscopy (FTIR), were utilized to comprehend the effects of g-C_3_N_4_ morphology and particle size on the physical and chemical characteristics of epoxy resin. Mechanical properties, such as tensile strength, strain, modulus, and fracture toughness, were determined for the composite samples. SEM analysis was performed to examine crack morphology in samples with different reinforcements. Findings indicate that optimal mechanical properties were achieved with a 0.5 wt% bulk g-C_3_N_4_ filler, enhancing tensile strength by 14%. SEM micrographs of fracture surfaces revealed a transition from brittle to rough morphology, suggesting increased toughness in the composites. While the TGA results showed no significant impact on degradation temperature, dynamic mechanical analysis demonstrated a 17% increase in glass transition temperature. Furthermore, the improvement in thermal breakdown up to 600 °C was attributed to reinforced covalent bonds between carbon and nitrogen, supported by FTIR results.

## 1. Introduction and Literature Review

The exponential expansion of manufacturing industries worldwide has triggered a pressing need for materials that not only offer enhanced mechanical properties but also address concerns related to environmental sustainability and cost efficiency. Over the past few decades, polymers have emerged as key substitutes for traditional materials like metals across various applications. This shift is attributed to several advantages that polymers offer, including ease of processing, lightweight nature, heightened productivity, and cost reduction [1]. Polymers used in composite manufacturing are. broadly classified into thermoplastics and thermosets based on their chemical bonding characteristics. Thermoplastic matrix materials consist of one- or two-dimensional molecules, enabling them to soften at elevated temperatures and regain stiffness upon cooling. In contrast, thermoset polymers are characterized by strong cross-linkages and undergo curing processes involving heat, pressure, and/or light irradiation. The inherent structure of thermoset polymers often results in superior performance in terms of strength and stiffness [2].

Polymer systems offer a versatile platform for enhancing characteristics like strength through the incorporation of both organic and inorganic fillers and fibers. Organic fillers, sourced from natural carbon-based compounds such as nanocrystalline cellulose, carbon nanotubes, graphene, and carbon fiber, find extensive applications across various disciplines, including bio-fabrication, aerospace, and manufacturing [3]. Conversely, inorganic fillers are commonly utilized in sectors like the food, pharmaceutical, and paper manufacturing industries to improve electrical conductivity [4]. The composition, type, quantity, dispersion, and interaction of fillers within the polymer matrix significantly influence reinforcement, enabling the production of a wide array of polymer composites with improved qualities for diverse industrial and technical applications [5]. Although significant progress has been made, issues such as brittleness, inadequate strength, poor stability at low temperatures, and low fracture toughness continue to present challenges [6].

Carbon nanomaterials are widely employed as reinforcing agents to improve the thermomechanical resistance, corrosion resilience, electrical conductivity, and flame-retardant attributes of polymer composites. This enhancement stems from their exceptional inherent properties and their ability to uniformly disperse within diverse polymeric matrices [7]. 

Rajsekhar et al. [8] conducted a study on the mechanical properties of epoxy resins with the addition of nano-alumina. They observed a notable enhancement in fracture toughness, which increased by 64% with 0.5 wt% nano-alumina samples. 

Wang et al. [9] proposed the use of graphene oxide as a 2D reinforcing agent to improve the mechanical properties of epoxy and improve the dispersion of carbon nanotubes. Tang et al. [10] investigated the impact of graphene dispersion on the mechanical properties of graphene/epoxy composites. Highly dispersed graphene enhances fracture toughness by 52% at 0.2 wt.% loading, compared to a 24% improvement for poorly dispersed graphene. Tensile and flexural moduli showed no significant differences with varying dispersion levels. This study highlighted the importance of the good dispersion of fillers for optimizing the mechanical performance of epoxy composites. Furthermore, Zeng et al. investigated the impact of self-assembled carbon nanotube montmorillonite on the mechanical properties of the epoxy matrix, revealing significant improvements in both strength and toughness [11,12].

Kumar et al. [13] prepared epoxy composites with graphene-like nanocarbon sheets (GNCs) at weight fractions between 0.005 and 2 wt%. The maximum tensile strength and tensile modulus were obtained at 0.1 wt% GNCs. The specific tensile modulus increased by 14.7%, from 536 MPa to 615 MPa, and the ultimate tensile strength increased by 3.6%, from 55 MPa to 57 MPa. Maximum flexural strength and flexural modulus were achieved at 1 wt% filler. The flexural strength increased from 2.28 GPa to 2.9 GPa, and the flexural modulus increased from 43.85 GPa to 56.42 GPa at 1 wt% of GNCs. The reinforcement effect at relatively low GNC weight fractions were attributed to good dispersion and chemical interaction.

Khan et al. [14] reviewed the polymer composites filled with various nitride compounds, emphasizing their potential applications in electronics and thermal management. It covers the structure and properties of nitride compounds, such as boron nitride, silicon nitride, carbon nitride, aluminum nitride, and titanium nitride, and their effects on polymer composites. These nitride compounds enhance the mechanical, thermal, and electrical properties of polymers, making them suitable alternatives to expensive and heavy metals like aluminum and copper. Among carbon nitrides, graphitic carbon nitride (g-C_3_N_4_) stands out as the most stable allotrope at room temperature, representing one of the earliest polymers reported in the literature [15]. The synthesis of g-C_3_N_4_ through the polycondensation of nitrogen-rich organic precursors such as di-cyandiamide, melamine, and thiourea has attracted significant attention [16]. Various synthetic methodologies have been proposed and the resulting product in terms of shape, morphology, and reactivity were explored [16,17].

Song et al. [18] found that the interlaminar shear stress was enhanced by up to 37.2% after the incorporation of g-C_3_N_4_ onto the surface of carbon fiber. Another study showed that the addition of g-C_3_N_4_ to epoxy resin for strengthening carbon fiber led to an increase in tensile strength by approximately 19.5% [19]. These findings collectively underscore the significant potential of various nanomaterials in augmenting the mechanical properties of polymer composites for diverse engineering applications.

This study investigates the influence of utilizing three distinct morphologies of g-C_3_N_4_—nanosheets, bulk, and nanotubes—as reinforcing agents on the thermomechanical properties of epoxy resin. The research entails the synthesis of these diverse g-C_3_N_4_ morphologies derived from melamine. Furthermore, the study explores the impact of varying g-C_3_N_4_ morphologies and different weight percentages on the static mechanical properties of the epoxy as well as its thermal stability. By analyzing these parameters, the study aims to elucidate the most effective morphology and optimal weight percentage of g-C_3_N_4_ for enhancing the thermomechanical properties of epoxy resin.

## 2. Methodology

### 2.1. Materials

The epoxy resin utilized in this study, Araldite LY 564, along with its corresponding hardener, Aradur 2954, were procured from Huntsman Inc. (Texas, USA). Melamine, with a purity of 99%, was obtained from Sigma-Aldrich (Massachusetts, USA). Additionally, various other materials and chemicals were employed, including nitric acid solution, ethanol, ethylene glycol, and distilled water, to facilitate the synthesis and processing of the composite materials.

### 2.2. Preparation Process of the Particles

#### 2.2.1. Bulk g-C_3_N_4_

A measure of 10 g of melamine was subjected to heating in a closed 30 mL ceramic crucible. The heating rate was maintained at 10 °C per minute, gradually increasing the temperature from 25 °C to 550 °C. This heating process was carried out in a well-ventilated furnace. Subsequently, once the temperature reached 550 °C, it was held constant for a duration of 4 h. This controlled heating and holding period facilitated the transformation of melamine into g-C_3_N_4_ powder (~7 g produced), as described in [20].

#### 2.2.2. g-C_3_N_4_ Nanotubes

A solution comprising 1 g of melamine dissolved in 30 mL of ethylene glycol was combined with 60 mL of a 0.1 M aqueous nitric acid solution at a temperature of 25 °C for a duration of 20 min. Afterward, the resultant solution underwent centrifugation and was subjected to three washes with ethanol to remove any residual nitric acid and ethylene glycol. Following this, the obtained white precipitate was dried under reduced pressure at 60 °C for a period of 12 h. Subsequent to the drying process, the material was heated to 400 °C at a rate of 5 °C per minute and held at this temperature for 2 h. This process resulted in the formation of a yellow powder, indicative of the presence of g-C_3_N_4_ nanotubes, as reported in [21].

#### 2.2.3. g-C_3_N_4_ Nanosheets

Nanosheets of g-C_3_N_4_ were synthesized through a hydrothermal treatment of melamine followed by calcination at elevated temperatures. Initially, 2 g of melamine was dispersed in 30 mL of water and stirred for 30 min. The resulting suspension was transferred to a 50 mL autoclave lined with Teflon and heated to 200 °C for a duration of 12 h. After cooling the white solid to room temperature, it was washed with distilled water and subsequently dried overnight under reduced pressure at 60 °C. The dried product was subsequently transferred into a 50 mL alumina crucible, which was fitted with a lid, and subjected to heating in a furnace at a rate of 2.3 °C per minute. It was maintained at a temperature of 550 °C for 4 h to form g-C_3_N_4_ nanosheets [22].

To insure uniformity among the three powders produced, the resulting yellow powder was crushed using a mortar and pestle before undergoing sieving to achieve a particle size of less than 38 mm, as detailed in [20].

### 2.3. Preparation of g-C_3_N_4_/Epoxy Composites

The required quantity of g-C_3_N_4_ for each experiment was determined based on a weight percentage relative to the combined mass of the polyamine hardener (Aradur 2594) and epoxy resin (Araldite LY 564).

Nine distinct sets of samples were prepared, each incorporating different weight percentages of g-C_3_N_4_ bulk, g-C_3_N_4_ nanosheets, and g-C_3_N_4_ nanotubes: specifically, 0.25%, 0.5%, and 1% by weight. The g-C_3_N_4_ powder was gradually introduced into a beaker containing 110 g of epoxy resin, followed by sonication for 30 min, and subsequent mixing at a speed of 10,000 rpm for 45 min. Due to the elevated mixing speed, the mixture experienced a rise in temperature, necessitating cooling to room temperature (RT) by placing the beaker on an ice pack. Once the mixture reached room temperature, 35.8 g of hardener was slowly added. Following degassing under vacuum at 25 °C for 2 h, the mixture was transferred into an aluminum mold and subjected to an additional 2 h of degassing at 25 °C. The samples were cured at 80 °C for 60 min, followed by post-curing at 140 °C for eight hours in accordance with the manufacturer’s instructions, as outlined in [20].

### 2.4. Characterization 

**Figure 1:**
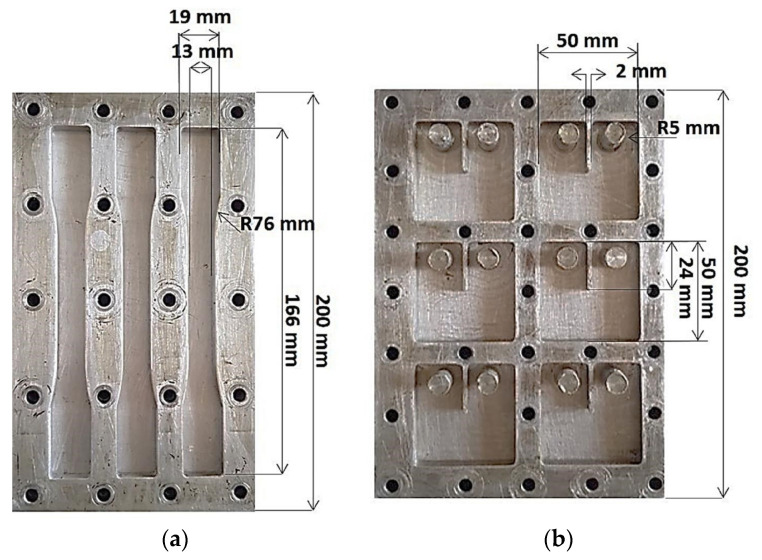
(**a**) Specifications of the molds used for fabricating the dog-bone samples intended for the mechanical tensile test and (**b**) dimensions of the mold utilized for the compact tension test [20].

To evaluate the static mechanical properties of the produced composites, tensile testing and compact tension testing methods were employed. Mechanical tensile testing was conducted, utilizing a universal testing machine (UTM) equipped with a 100 kN load cell, operating at a loading speed of 1 mm/min using ASTM D638 standard test procedure. A laser extensometer was used to measure the strain. The aluminum mold used for preparing the tensile specimens had a gauge length of approximately 50 mm and a cross-sectional area of 13 × 7 mm^2^ (see Figure 1a). From each batch, at least 5 samples were tested and analyzed [20]. The elastic modulus and the stress at failure were key parameters analyzed from the test results.

The compact tension of the materials was completed using the same UTM, equipped with a 100 kN load cell, and operated at a cross-head speed of 0.5 mm/min. Square samples were prepared with dimensions of 40 mm in width, 10 mm in thickness, and featuring a 24 mm notch positioned at the center of the sample, using the ASTM standard D5045 (refer to Figure 1b). To conduct the test, a 1 mm crack was initiated at the tip of the notch using a blade. The results of the compact tension test were evaluated using the following equation derived from ASTM D5045 to determine the plane–strain fracture toughness, KI (in MPa.m^0.5^):

$$K_{I}=\frac{P_{Q}}{B\sqrt{W}}\times \frac{\left(2+\alpha \right)}{{\left(1-\alpha \right)}^{\frac{3}{2}}}\times (0.866+4.64\;\alpha -13.32\alpha^{2}+14.72\alpha^{3}-5.6\alpha^{4})$$

The equation involves parameters such as the sample’s width (W) and thickness (B), crack length to sample width ratio (α), and the change between the maximum and minimum load (ΔP) in MN.

Following tensile testing, the crack morphology of the samples was observed using a Tescan MIRA3 Field Emission Scanning Electron Microscope (FESEM). The samples were coated with a 15 nm thick layer of platinum prevent charging. Similarly, the as-prepared g-C_3_N_4_ samples were examined using the same SEM microscope by adding a small amount of the power to a stud with carbon tape. The samples were also coated with platinum to avoid charging. All samples were tested at a voltage of 10 kV using a secondary electron detector.

Dynamic light scattering (DLS) with the Q2000 instrument was employed to ascertain the particle size of the three variants of g-C_3_N_4_ (dispersed using different solvents). Specifically, a concentration of 1000 mg/L of bulk g-C_3_N_4_, nanotubes, and nanosheets were dispersed in two distinct solutions (ethanol and DMF) to identify the most suitable dispersing agent. Following the selection of the optimal dispersing solution, two concentrations of filler were sonicated for 3 min and subsequently analyzed using DLS at three different time intervals (t_1_ = 0 min, t_2_ = 15 min, and t_3_ = 2 h), with four measurements conducted and averaged for each mixture.

The glass transition temperature of the epoxy composites was determined using a Q100 DSC (TA Instruments, New Castle, Delaware, United States) in a heat/cool/heat mode with heating and cooling rates set at 10 °C/min from −50 °C to 200 °C. Glass transition temperatures were calculated based on fluctuations in heat flow observed during the 3rd cycle (heating, cooling, and heating again).

To measure the thermal stability of the epoxy composites, TGA Q500 (TA Instruments) was used with nitrogen as the purge gas. Analysis was completed over a temperature range of 25 to 800 °C at a rate of 10 °C/min, using samples weighing between 7 mg and 15 mg. The decomposition temperature (T_d_) was determined from the onset temperature observed in the obtained thermographs.

Furthermore, Fourier transform infrared (FTIR) spectroscopy was conducted on melamine (the starting material) and the synthesized g-C_3_N_4_ (bulk, nanotubes, and nanosheets) using an Agilent cary 630 FT-IR instrument (Santa Clara, CA, USA) in ATR mode.

## 3. Results and Discussion

### 3.1. Characterization of the Synthesized Particles

The synthesized particles underwent various analyses and characterizations to elucidate their physical–chemical properties and to discern differences in morphology and particle size among the different forms of g-C_3_N_4_. SEM, DLS, TGA, and FTIR were employed to perform the characterization.

**Figure 2:**
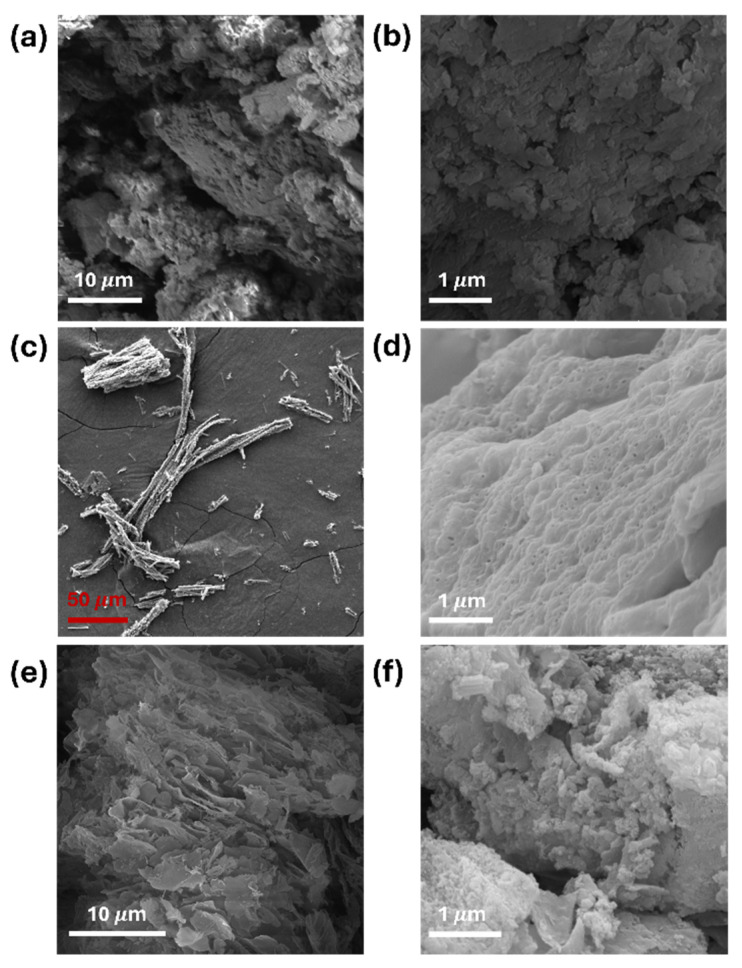
SEM micrographs of g-C_3_N_4_ (**a**,**b**) bulk, (**c**,**d**) nanotubes, and (**e**,**f**) nanosheets.

SEM imaging of bulk g-C_3_N_4_, g-C_3_N_4_ nanosheets, and g-C_3_N_4_ nanotubes revealed substantial variations in morphology, as depicted in Figure 2. While pristine melamine particles ere known for their stone-like morphology, upon heating, melamine, a trimer of cyanamide, undergoes pyrolysis and polymerization to yield g-C_3_N_4_, resulting in the formation of flaky sheet-like structures stacked together (Figure 2a,b), as reported in the literature [23,24].

The morphology of g-C_3_N_4_ nanotubes exhibited structures characterized by open ends, as illustrated in Figure 2c,d [25]. Additionally, the synthesized g-C_3_N_4_ samples derived from melamine underwent transformation into 2D sheet like structures (Figure 2e,f), displaying a typical nanosheet structures with smooth surfaces, consistent with findings in the literature [22]. The TEM images of the g-C_3_N_4_ nanotubes and nanosheets are available in other papers [21,22], from which we have also adopted the synthesis methods.

Our preliminary investigation revealed that g-C_3_N_4_ demonstrates superior dispersion in dimethylformamide (DMF) compared to ethanol (refer to Figure S1). Table 1 presents the typical particle size distribution of the three g-C_3_N_4_ variants (nanosheets, bulk, and nanotubes) dispersed in DMF at various time intervals, as determined by DLS analysis. As indicated in Table 1, after 2 h, the size of g-C_3_N_4_ bulk and g-C_3_N_4_ nanosheets reaches a minimum value of 3.05 µm and 1.6 µm, respectively, while the size of g-C_3_N_4_ nanotubes remains largely unchanged. It is important to note that the observed average hydrodynamic diameter of the three forms of g-C_3_N_4_ from DLS analysis may be affected by multiple scattering effects, leading to potential errors [25].

DLS quantifies the timescale of light fluctuations reaching the detector resulting from the random diffusion/movement of suspended particles within a sample cuvette. Smaller particles exhibit rapid diffusion, resulting in fast intensity fluctuations, while larger particles diffuse more slowly, leading to slower variations in scattered light intensity. In cases of multiple scattering, a photon of light is scattered by a diffusing particle, then re-scattered by one or more particles before reaching the detector, which can distort the observed particle size [26]. It is important to note that the size measured using DLS refers to the size of the agglomerates in the suspension rather than individual nanosheets or nanotubes.

The decrease in average particle size distribution over time can be attributed to the sedimentation of larger particles in the cuvette, leaving smaller particles dispersed for a longer duration. Consequently, DLS detects smaller particles more prominently over time, thus explaining the reduction in particle size observed for g-C_3_N_4_ bulk and g-C_3_N_4_ nanosheets. In the case of g-C_3_N_4_ nanotubes, their hollow structure enables them to float in the solution for longer periods of time. At higher concentrations (0.25 g/L), slight aggregation of the nanotubes (driven upwards) causes an increase in the detected size (4.9 ± 0.1 µm). For the following duration of 45 min, some of the nanotubes settled at the bottom of the vial causing the measured DLS size to drop back to 3.1 ± 0.1 µm. In spite of this decrease, the stability of the nanotubes in suspension proved superior to g-C_3_N_4_ fillers in bulk and as nanosheets.

**Figure 3:**
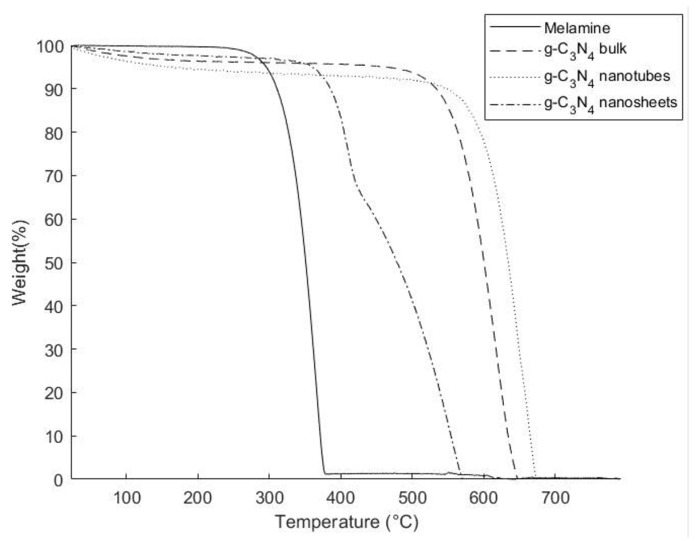
TGA thermograms of pure melamine, g-C_3_N_4_ bulk, g-C_3_N_4_ nanosheets, and g-C_3_N_4_ nanotubes.

Figure 3 illustrates the TGA curves thermograms of pure melamine, g-C_3_N_4_ bulk, g-C_3_N_4_ nanotubes, and g-C_3_N_4_ nanosheets. Pure melamine undergoes decomposition and sublimation at around 305 °C without any reduction in the weight prior to that temperature. This indicated that melamine did not undergo any polymerization reaction in that temperature range and reinforces the need to perform the synthesis of g-C_3_N_4_ in a closed-lid crucible to avoid this sublimation. On the other hand, the TGA curves thermograms of g-C_3_N_4_ bulk, g-C_3_N_4_ nanosheets, and g-C_3_N_4_ nanotubes indicated a slight weight loss of 2.5% within the range of 25–200 °C. While the weight loss below 100 °C is attributed to the desorption of water molecules on the surface, the change in weight above this temperature was an indication of additional polymerization taking place of the intermediates (melem and melan). This condensation reaction led to the loss of nitrogen and hydrogen from the samples in the form of ammonia gas. However, the direct thermal decomposition of g-C_3_N_4_ bulk leads to a significant weight loss of 97.5% between 600 °C and 760 °C, similar to that observed for nanotubes. At this temperature, covalent bonds between carbon and nitrogen begin to break leading to the full decomposition of the organic filler. This enhancement is typically attributed to the polymerization of melamine trimer, leading to the formation of extended 2D chains of g-C_3_N_4_. Notably, g-C_3_N_4_ nanosheets exhibited rapid disintegration without any residue within the range of 430–760 °C. The lower decomposition temperature of g-C_3_N_4_ nanosheets was attributed to the lower amount of NH_2_ and NH groups, resulting in fewer carbon and nitrogen bonds [27].

**Figure 4:**
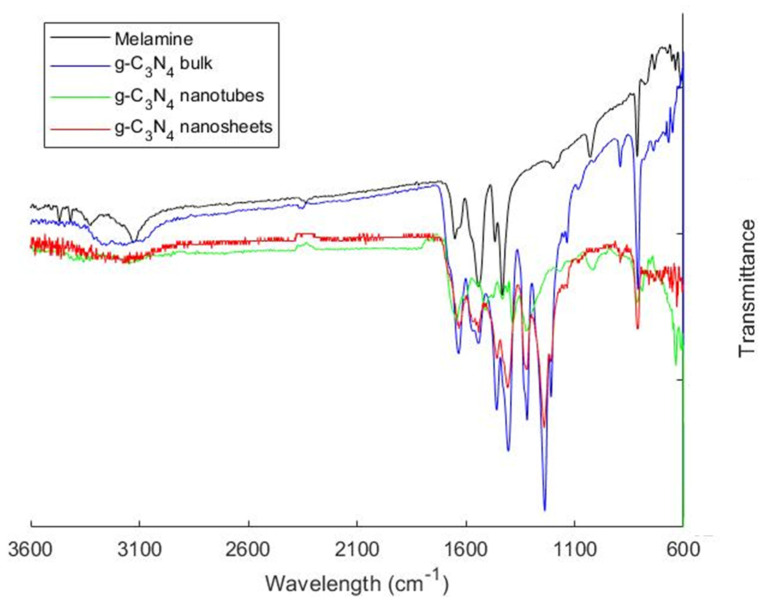
FTIR spectra of pure melamine, g-C_3_N_4_ bulk, g-C_3_N_4_ nanosheets, and g-C_3_N_4_ nanotubes.

The FTIR spectrum for melamine (Figure 4) reveals three main absorption regions in the chemical structures. Clear N-H stretching vibration bands were observed within 3300 and 3000 cm^−1^ and within 1650 and 1580 cm^−1^, while peaks between 1020 and 1200 cm^−1^ correspond to the C-N bending vibration of amines, indicative of vibrations in melamine’s aromatic rings, as evidenced by the peak around 810 cm^−1^. A comparison with the one that is specific to the produced g-C_3_N_4_ bulk revealed that previously sharp and strong absorption peaks in the region of 3000–3600 cm^−1^ had merged into a broad peak centered at around 3100 cm^−1^, corresponding to the NH or NH_2_ stretching vibrations, suggesting the calcination of existing amino groups in melamine upon pyrolysis. Other signals were observed in the 1100–1600 cm^−1^ region, indicating the condensation of the triazine rings [27].

A shift in the major signals between g-C_3_N_4_ bulk and g-C_3_N_4_ nanotubes was also noted. The appearance of a signal at around 1600 cm^−1^ could be assigned to the C=N stretching vibration of the inherent structure of g-C_3_N_4_ nanotubes. Vibrations at 1641 cm^−1^, 1710 cm^−1^, and 3352 cm^−1^ could be assigned to the functionalized g-C_3_N_4_ nanotubes (carbonyl, carboxyl, and hydroxyl functional groups), as reported in the literature [28]. The chemical structure of g-C_3_N_4_ bulk and g-C_3_N_4_ nanosheets were presumed to be similar since their FTIR spectra were identical. Peaks related to the N-H stretch of amino groups, aromatic C-N stretching modes of the s-triazine ring were observed at 3000–3500 cm^−1^ and 1200–1700 cm^−1^, respectively [23]. Peaks between 1200 and 1700 cm^−1^ for g-C_3_N_4_ nanosheets appeared sharper than those for g-C_3_N_4_ bulk powder, suggesting a more ordered packing of the s-triazine motifs in the nanosheet layers [24].

### 3.2. Static Mechanical Properties and Fracture Morphology 

The mechanical properties of epoxy composites incorporating various types of g-C_3_N_4_ at various concentrations were evaluated, encompassing tensile and compact tension tests. Table 2 summarizes the average tensile strength, strain, and modulus values, while Table S1 lists the KI values.

**Figure 5:**
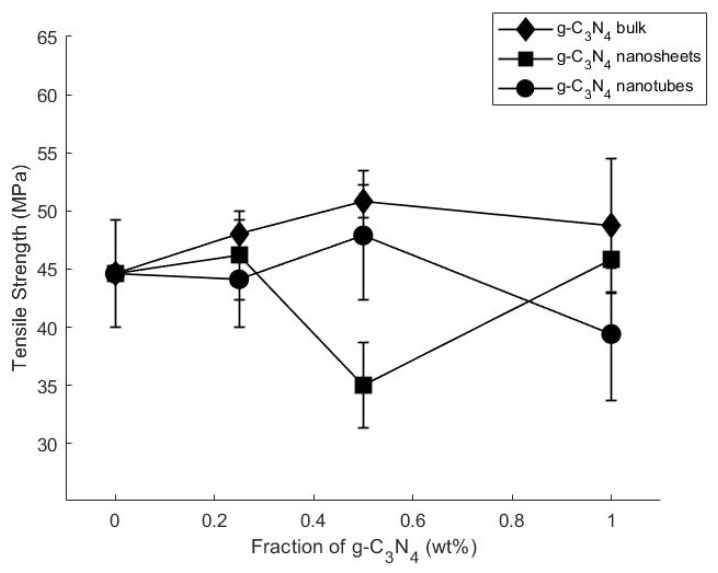
The average tensile strength of g-C_3_N_4-reinforced epoxy_ composites plotted against filler concentration (wt.%).

Figure 5 and Table 2 illustrate that the incorporation of g-C_3_N_4_ bulk and g-C_3_N_4_ nanotube particles led to an increase in the composites’ tensile strength. Notably, at 0.5 wt.% of g-C_3_N_4_ bulk and g-C_3_N_4_ nanotubes, the tensile strength peaked at 14% and 8%, respectively. However, a gradual decline in mechanical properties was observed beyond this threshold, particularly evident at 1 wt.% g-C_3_N_4_ bulk and g-C_3_N_4_ nanotubes–epoxy composites, hinting at filler agglomeration, as reported in prior studies [27]. It is possible that the strong interactions between NH_2_ and NH groups in g-C_3_N_4_ and the hydroxyl and epoxide groups of the resin contributed to these improvements.

Introducing g-C_3_N_4_ nanosheet particles exhibited a distinct trend. While strength initially improved by 3% with 0.25 wt% and 1 wt% addition of g-C_3_N_4_ nanosheets, it sharply declined to −21% with a 0.5 wt% addition, suggesting hindered interactions between the filler and the matrix, possibly due to the formation of large g-C_3_N_4_ agglomerates.

Moreover, increasing the concentration of g-C_3_N_4_ bulk and g-C_3_N_4_ nanotubes led to a reduction in the modulus of elasticity, from 853 MPa of pure epoxy to 768 MPa for g-C_3_N_4_ bulk, 803 MPa for g-C_3_N_4_ nanosheets, and 755 MPa for g-C_3_N_4_ nanotubes–epoxy. This discrepancy may be attributed to the density difference, as g-C_3_N_4_ nanotubes and g-C_3_N_4_ nanosheets are smaller and less dense, potentially leading to increased overall agglomeration. It is important to note that these nanoparticles tend to aggregate when mixed with the hardener, and such aggregates may not uniformly disperse in the composite matrix, further influencing the mechanical response of the resulting composites.

Table S1 reveals that there was no enhancement in fracture toughness in any of the scenarios. Incorporating g-C_3_N_4_ bulk resulted in either a decrease (up to 16%) or no alteration in fracture toughness. Likewise, the fracture toughness of g-C_3_N_4_ nanosheets and g-C_3_N_4_ nanotube composites declined, with a maximum drop of 25% observed upon increasing the weight percentage. This decline in fracture toughness can be attributed to the type of crack formation. Previous studies have identified two distinct types of crack propagation: one involving continuous propagation with minimal fracture tip deformation (0.5–2 μm), and the other characterized by crack growth in a stick–slip manner. Consequently, the same material might exhibit both types of fracture behavior [29]. The mechanisms governing toughening are influenced by numerous factors such as the type, size, volume fraction, and particle–matrix bonding. The configurations of the inclusions, whether single or clustered, and their uniform dispersion within the matrix, also impact the material’s fracture toughness [30].

**Figure 6:**
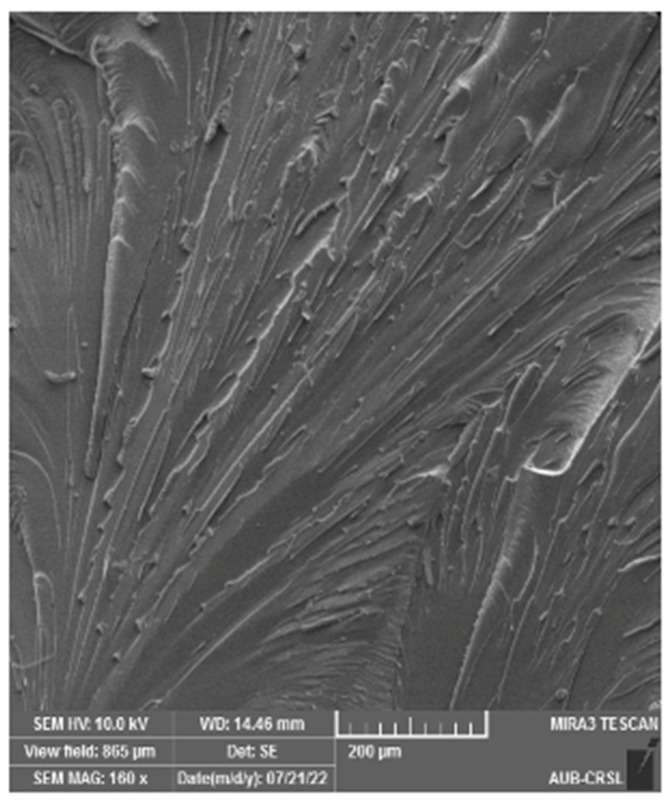
SEM micrograph of the fractured epoxy sample after tensile testing.

**Figure 7:**
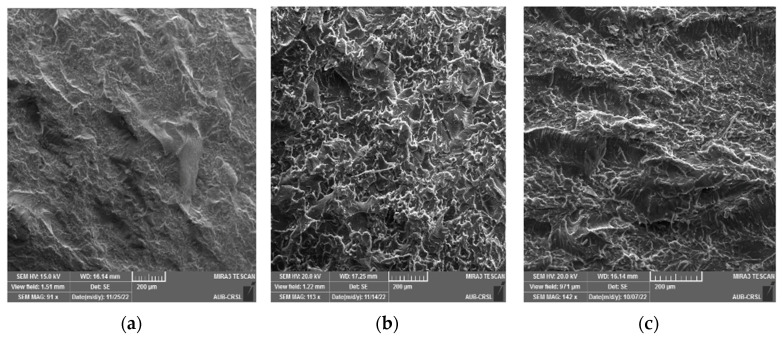
SEM micrograph of the fractured epoxy composite reinforced with g-C_3_N_4_ bulk: (**a**) 0.25 wt%, (**b**) 0.5 wt%, (**c**) 1 wt%.

**Figure 8:**
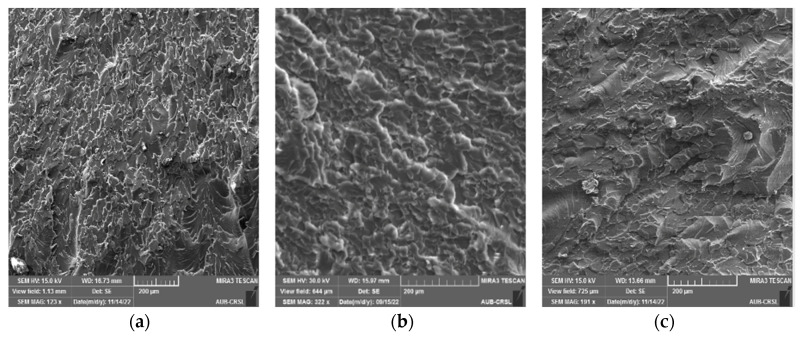
SEM micrograph of the fractured epoxy composite reinforced with g-C_3_N_4_ nanosheets: (**a**) 0.25 wt%, (**b**) 0.5 wt%, (**c**) 1 wt%.

**Figure 9:**
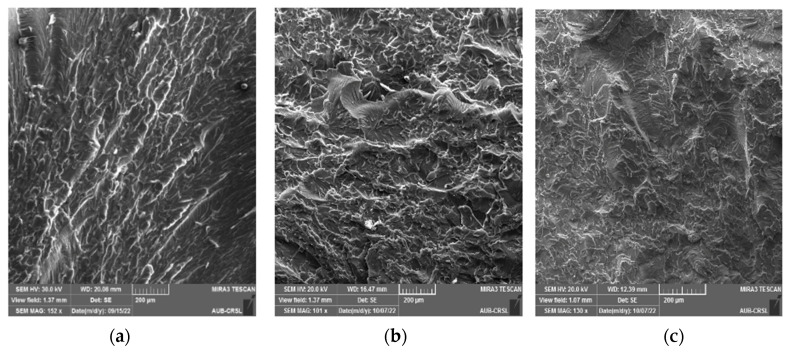
SEM micrograph of the fractured epoxy composite reinforced with g-C_3_N_4_ nanotubes: (**a**) 0.25 wt%, (**b**) 0.5 wt%, (**c**) 1 wt%.

SEM analysis was conducted on the cracked surface (of the samples) to gain deeper insights into the outcomes of mechanical testing, as illustrated in Figure 6, Figure 7, Figure 8 and Figure 9. Pure epoxy exhibited distinctive brittle behavior, with the crack propagating smoothly and directly from the bottom left corner to the other corner with minimal deviation (Figure 6). Similar behavior was observed in samples containing 0.25 wt% of g-C_3_N_4_ bulk, g-C_3_N_4_ nanosheets, and g-C_3_N_4_ nanotubes (Figure 7, Figure 8 and Figure 9a), as well as in samples with 0.5 wt% of g-C_3_N_4_ nanosheets (Figure 8b). In all these cases, the fracture path was distinct and linear, indicates brittle behavior and weak interactions between g-C_3_N_4_ and epoxy.

However, the addition of 0.5 wt% of g-C_3_N_4_ bulk and g-C_3_N_4_ nanotubes resulted in a significantly rougher crack surface with an ambiguous crack trajectory. Due to the potential deflection of fractures by particles, multiple cracks were observed, extending in various directions. This observation suggests that the enhancement in mechanical properties with 0.5 wt% of g-C_3_N_4_ bulk and g-C_3_N_4_ nanotube particles is attributed to increased strain energy consumption and improved interfacial bonding strength between the filler and matrix. Achieving a homogeneous dispersion of the filler at 0.5 wt% led to improved mechanical properties, whereas filler loadings below 0.5 wt% did not yield significant enhancements in the matrix [20].

For samples with 1 wt%, a similar rough fracture morphology was observed; however, there is clear evidence of nanofiller agglomeration (as shown in Figure 7c, Figure 8c and Figure 9c). The increase in wt% of particles led to the formation of island-like structures, rich in filler within the resin, consistently with the literature [9,31]. These regions containing g-C_3_N_4_ agglomerates act as system flaws, promote the growth of additional cracks, and contribute to the premature failure of the test specimen by creating areas of higher stress concentration where cracks may initiate [32].

### 3.3. Thermal Properties

The thermal degradation behavior of epoxy composites reinforced with various types of g-C_3_N_4_ was investigated through TGA analysis. Figures S2–S4 show the TGA thermograms of the obtained composites and Table 3 provides a summary of the results. The average degradation temperature (Td) of pure epoxy resin was found to be 396.1 ± 0.4 °C, with a residue content of about 1.12% at 800 °C, in agreement with the literature [20]. Once reinforced, the decomposition profiles of the epoxy composites showed similar Td values ranging between 399.4 and 401.7 °C. This observed increase in decomposition temperature could be attributed to the so-called “pyrolysis barrier” of g-C_3_N_4_, which prevents the dispersion of gaseous molecules generated during the decomposition of the epoxy matrix. However, the increase in filler concentration did not significantly affect the Td values. The residual polymer concentration at 800 °C was unaffected by the addition of fillers. This was predicted from Figure 3, showing complete degradation of fillers before 800 °C. Interesting to note that in comparison to pure epoxy resin, all the reinforced composites demonstrated a lower rate of decomposition before the Td. As can be observed in the thermograms of Figures S2–S4, while the decomposition of all samples began at around 100 °C, at Td the observed weight of the samples was approximately 7% higher for all composites. Similarly, a decrease in the rate of decomposition was observed for the composites after 50% of the original weight was lost. These observations were an indication of an overall improvement in thermal stability due to stronger covalent bonding between the g-C_3_N_4_ filler and epoxy resin that prevented the rapid decomposition of the composites. It was concluded that a low concentration of the filler (up to 1 wt%) cannot enhance the thermal degradation behavior of the epoxy composites.

DSC measurements were used to determine the glass transition temperatures (Tg), with corresponding thermograms provided in Figures S5–S7, and average Tg values summarized in Table 4. The introduction of fillers increased the Tg value, indicating improved thermal stability. No evidence of endothermic or exothermic peaks was observed in the tested temperature range tested, indicating the complete cure behavior of the composites. The Tg of pure epoxy (132 °C) increased with filler addition, reaching a maximum increase of about 17% for 1 wt% of g-C_3_N_4_ bulk. This could be attributed to the covalent bonds connections between the fillers and the epoxy matrix, which reduce matrix mobility and result in a larger shift in Tg. The -NH_2_ groups on the surface of g-C_3_N_4_ particles can potentially react with epoxy monomers during polymerization [33].

## 4. Conclusions

This study explored using different forms of graphitic carbon nitride (g-C_3_N_4_) to enhance epoxy resin composites. By varying the concentration and shape of the filler, we assessed their impact on the composite’s strength. Techniques like microscopy revealed that adding 0.5 wt% bulk g-C_3_N_4_ increased composite strength by 14%. Fractured surfaces showed a transition from brittle to rough, indicating potential toughness enhancements due to an improvement in chemical boding between the epoxy matrix and g-C_3_N_4_ fillers. This was also reflected by the increase in glass transition temperature by up to 17%. Our work addresses the lack of literature on the effects of g-C_3_N_4_ morphology on epoxy composites. We are among the few to demonstrate how these morphologies influence mechanical properties, suggesting mechanisms where filler shape and dispersion affect stress distribution and crack propagation. Achieving uniform filler distribution remains a challenge, highlighting the need for ongoing research to optimize filler–matrix interactions. Additionally, consistent monitoring of epoxy composites is crucial due to their susceptibility to environmental factors. In summary, our study offers fundamental insights into the role of g-C_3_N_4_ morphology in epoxy composites, paving the way for future research to develop more effective composite materials.

## Figures and Tables

**Table 1:** DLS measurements to determine the average particle size of g-C_3_N_4_ bulk, g-C_3_N_4_ nanotubes, and g-C_3_N_4_ nanosheets at various concentrations and time intervals.

Time (min)		t_1_ = 0		t_2_ = 15		t_3_ = 60	
Concentration (g/L)		0.25	0.125	0.25	0.125	0.25	0.125
**Size (µm)**	**g-**C_3_N_4_**Bulk**	9.6 ± 0.1	9.3 ± 0.1	9.2 ± 0.2	9.2 ± 0.09	3.1 ± 0.09	2.8 ± 0.06
	**g-**C_3_N_4_ **Nanotubes**	3.7 ± 0.07	3.3 ± 0.07	4.9 ± 0.1	3.4 ± 0.06	3.1 ± 0.1	3.0 ± 0.1
	**g-**C_3_N_4_ **Nanosheets**	5.7 ± 0.08	4.2 ± 0.09	5.1± 0.06	3.9 ± 0.08	1.7 ± 0.06	1.9 ± 0.03

**Table 2:** Tensile strength, strain, and modulus of elasticity of g-C_3_N_4-reinforced epoxy_ composites.

Composite	Filler Concentration wt%	Tensile Strength (MPa)	% Change	Elastic Modulus (MPa)	% Change	Strain (mm/mm)	% Change
**Pure Epoxy**	0	44.61 ± 4.6	-	853.6 ± 83.6	-	0.07 ± 0.002	-
**g-**C_3_N_4_ **bulk**	0.2	48.04 ± 1.23	7	883.4 ± 11.5	3	0.07 ± 0.002	0
	0.5	50.82 ± 1.45	14	830.7 ± 76.3	−2	0.06 ± 0.009	−14
	1.0	48.73 ± 5.8	9	768.8 ± 95.4	−9	0.07 ± 0.002	0
**g-**C_3_N_4_**nanosheets**	0.25	46.19 ± 3.8	3	854.08 ± 22.5	0	0.09 ± 0.006	18
	0.5	35 ± 3.7	−21	983.5 ± 66.8	15	0.05 ± 0.006	−29
	1.0	45.82 ± 2.81	3	803.5 ± 38.2	−5	0.07 ± 0.002	0
**g-**C_3_N_4_**nanotubes**	0.25	44.12 ± 4.08	−1	921.7 ± 55	8	0.07 ±0.002	0
	0.5	47.896 ± 5.55	8	956.8 ± 151	12	0.07 ± 0.002	0
	1.0	39.41 ± 5.7	−11	755.64 ± 13.2	−11	0.06 ± 0.004	−14

**Table 3:** The average degradation temperature (Td) and the residue % of g-C_3_N_4_-reinforced epoxy composites.

Composite	Filler Concentration wt%	Average Residue (%)	Average Td (°C)
**Pure Epoxy**	0	1.12	396.1 ± 0.4
**g-**C_3_N_4_ **bulk**	0.25	1.14	401.7 ± 0.1
	0.5	1.38	401.3 ± 0.5
	1.0	1.17	402.9 ± 0.8
**g-**C_3_N_4_ **nanosheets**	0.25	1.24	400.1 ± 0.4
	0.5	1.77	400.5 ± 0.9
	1.0	1.72	401.6 ± 0.3
**g-**C_3_N_4_ **nanotubes**	0.25	1.63	398.7 ± 0.3
	0.5	1.21	399.6 ± 0.6
	1.0	1.36	399.4 ± 0.4

**Table 4:** The average glass transition temperature (Tg) of g-C_3_N_4_-reinforced epoxy composites.

Composite	Filler Concentration wt%	Average Onset Tg (°C)
**Pure Epoxy**	0	132 ± 0.44
**g-**C_3_N_4_ **bulk**	0.25	152 ± 0.21
	0.5	153 ± 0.33
	1.0	155 ± 0.43
**g-**C_3_N_4_ **nanosheets**	0.25	153 ± 0.57
	0.5	154 ± 0.36
	1.0	151 ± 0.41
**g-**C_3_N_4_ **nanotubes**	0.25	145 ± 0.39
	0.5	149 ± 0.52
	1.0	152 ± 0.45

## Data Availability

Data are contained within the article and Supplementary Materials.

## Supplementary Materials

The following supporting information can be downloaded at: https://www.mdpi.com/article/10.3390/polym16131935/s1, Figure S1: Comparison between the 2 solutions. [1]: g-C_3_N_4_ bulk in DMF; [2]: g-C_3_N_4_ nanotubes in DMF; [3]: g-C_3_N_4_ nanosheets in DMF; [4]: g-C_3_N_4_ bulk in ethanol; [5]: g-C_3_N_4_ nanotubes in ethanol; [6]: g-C_3_N_4_ nanosheets in ethanol. Figure S2: TGA curves for pure epoxy and the manufactured composites with the g-C_3_N_4_ bulk. Figure S3: TGA curves for pure epoxy and the manufactured composites with the g-C_3_N_4_ nanosheets. Figure S4: TGA curves for pure epoxy and the manufactured composites with the g-C_3_N_4_ nanotubes. Figure S5: DSC graphs for pure epoxy and the manufactured composites with the g-C_3_N_4_ bulk. Figure S6: DSC graphs for pure epoxy and the manufactured composites with the g-C_3_N_4_ nanosheets. Figure S7: DSC graphs for pure epoxy and the manufactured composites with the g-C_3_N_4_ nanotubes. Table S1: The average and improvement values of the fracture toughness of g-C_3_N_4_ bulk, g-C_3_N_4_ nanosheets, and g-C_3_N_4_ nanotubes-Epoxy composites with different concentrations.
