# Supplementary material for: Various Morphologies of Graphitic Carbon Nitride (g-C3N4) and Their Effect on the Thermomechanical Properties of Thermoset Epoxy Resin Composites
Source: Polymers (Basel). 2024 Jul 6;16(13):1935. doi: 10.3390/polym16131935 (PMC11243981; doi:10.3390/polym16131935)
Supplement: Supplementary file 1 [file polymers-16-01935-s001.zip › polymers-3003142-supplementary.pdf]

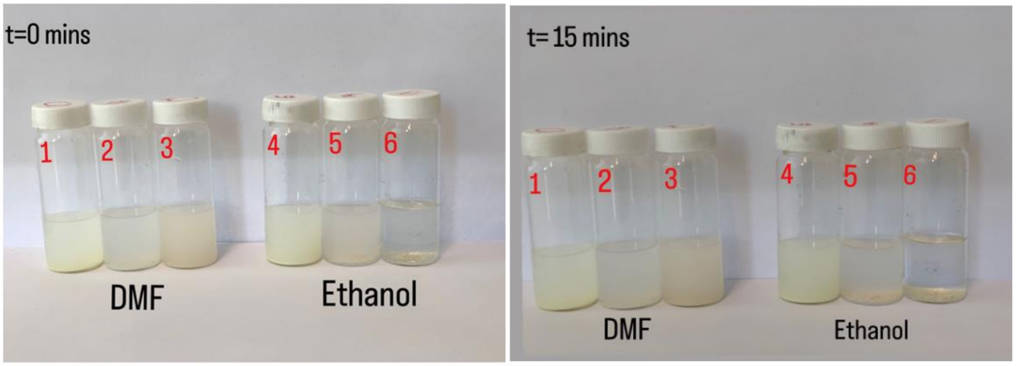

(a) (b)

Figure S1. Comparison between the 2 solutions. [1]: g-C_3N_4 bulk in DMF; [2]: g-C_3N_4 nanotubes in DMF; [3]: g-C_3N_4 nanosheets in DMF; [4]: g-C_3N_4 bulk in ethanol; [5]: g-C_3N_4 nanotubes in ethanol; [6]: g-C_3N_4 nanosheets in ethanol.

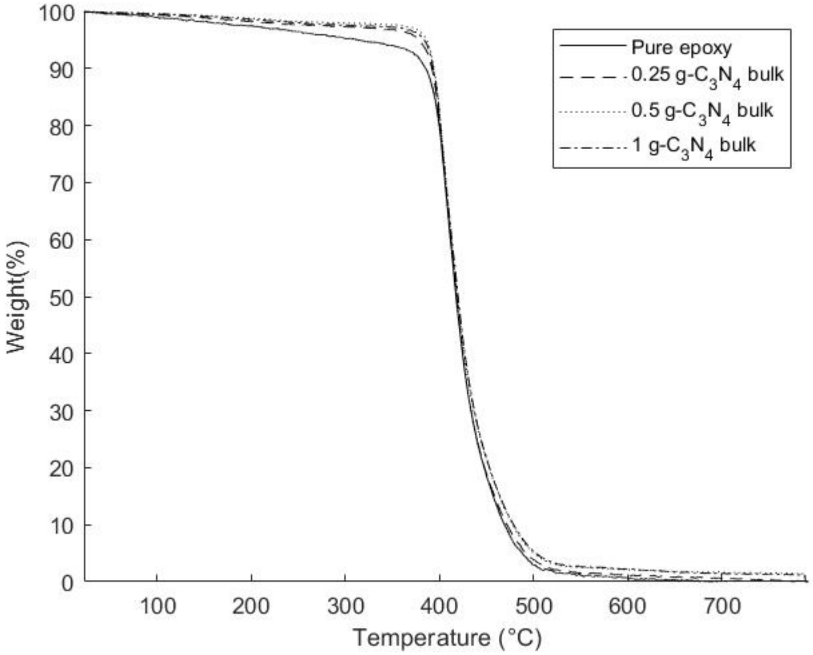

Figure S1. TGA curves for pure epoxy and the manufactured composites with the g-C_3N_4 bulk.

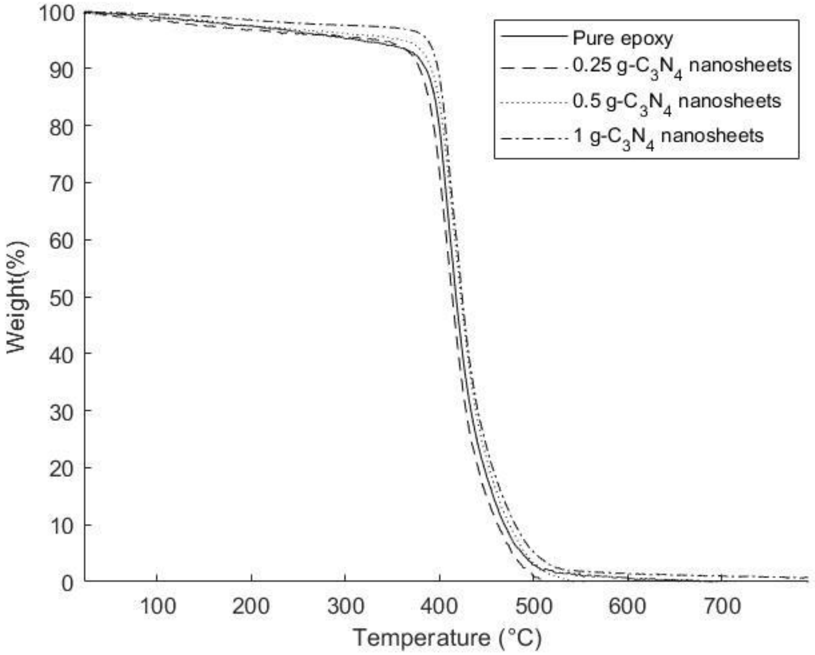

Figure S2. TGA curves for pure epoxy and the manufactured composites with the $g\text{-}C_3N_4$ nanosheets.

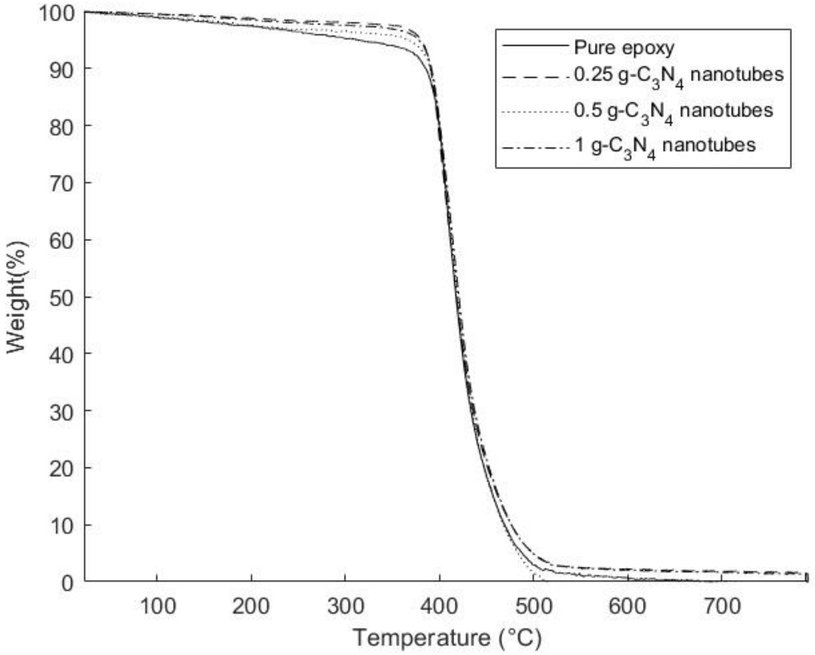

Figure S3. TGA curves for pure epoxy and the manufactured composites with the $g\text{-}C_3N_4$ nanotubes.

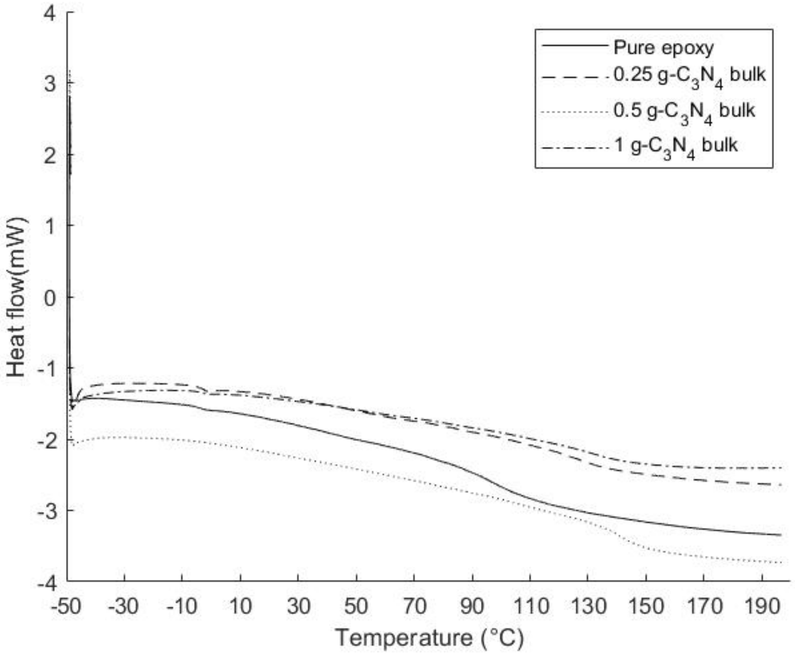

Figure S4. DSC graphs for pure epoxy and the manufactured composites with the g-C_3N_4 bulk.

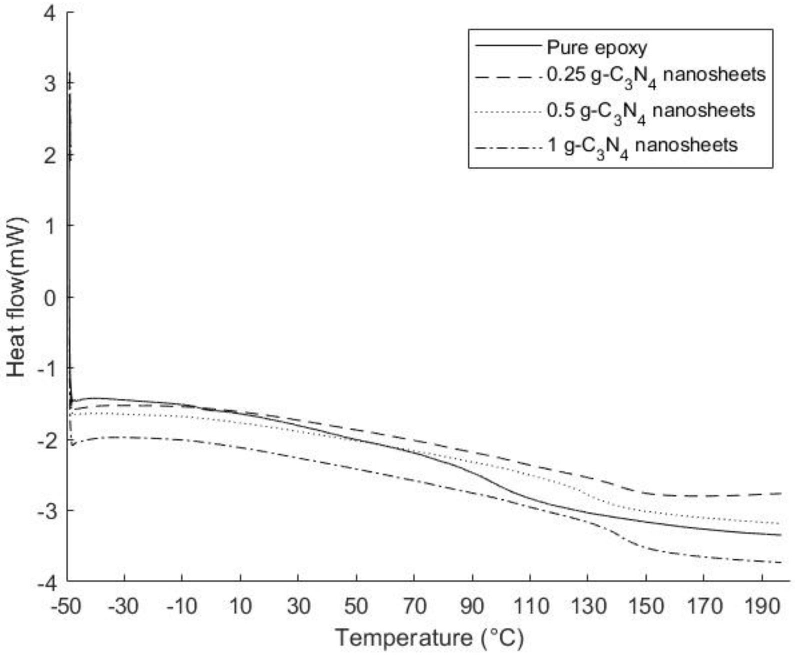

Figure S5. DSC graphs for pure epoxy and the manufactured composites with the g-C_3N_4 nanosheets.

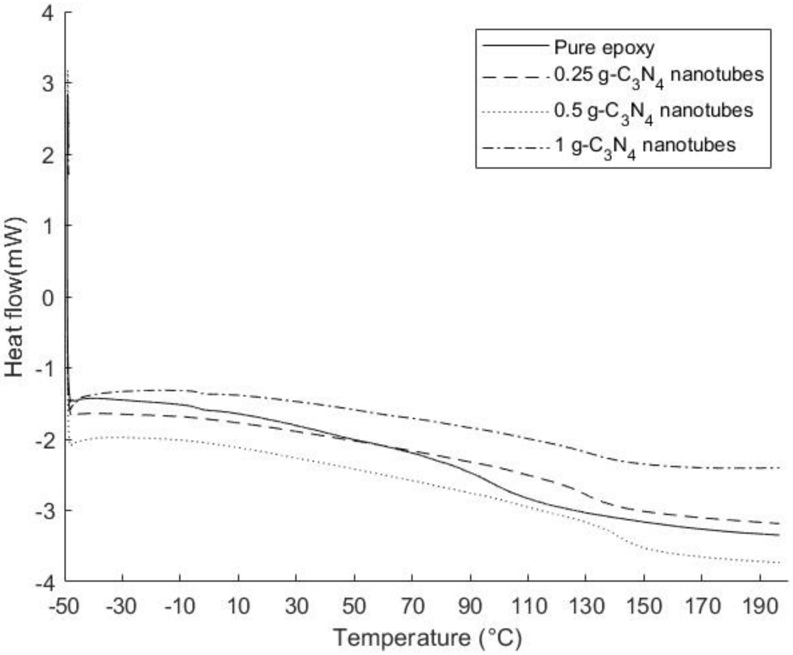

Figure S6. DSC graphs for pure epoxy and the manufactured composites with the $g\text{-}C_3N_4$ nanotubes.

Table S1. The average and improvement values of the fracture toughness of $g\text{-}C_3N_4$ bulk, $g\text{-}C_3N_4$ nanosheets, and $g\text{-}C_3N_4$ nanotubes-Epoxy composites with different concentrations.

Fraction of the particle (wt%)	Fracture toughness average with filler ($MN.m^{0.5}$)	% Change
**Pure poxy**	0.971 ± 0.05	-
**0.25 $g\text{-}C_3N_4$ bulk**	0.811 ± 0.02	-16
**0.25 $g\text{-}C_3N_4$ nanosheets**	0.971 ± 0.02	0
**0.25 $g\text{-}C_3N_4$ nanotubes**	0.86 ± 0.04	-11
**0.5 $g\text{-}C_3N_4$ bulk 0.5**	0.821 ± 0.07	-15
**0.5 $g\text{-}C_3N_4$ nanosheets**	0.957 ± 0.04	-1
**0.5 $g\text{-}C_3N_4$ nanotubes**	0.78 ± 0.02	-19
**1 $g\text{-}C_3N_4$ bulk**	0.969± 0.069	0
**1 $g\text{-}C_3N_4$ nanosheets**	0.72 ± 0.04	-26
**1 $g\text{-}C_3N_4$ nanotubes**	0.73 ± 0.007	-24
